# Identification of Binding Regions of Bilirubin in the Ligand-Binding Pocket of the Peroxisome Proliferator-Activated Receptor-A (PPARalpha)

**DOI:** 10.3390/molecules26102975

**Published:** 2021-05-17

**Authors:** Darren M. Gordon, Stephen H. Hong, Zachary A. Kipp, Terry D. Hinds

**Affiliations:** 1 Department of Neurosciences, University of Toledo College of Medicine and Life Sciences, Toledo, OH 43614, USA; Darren.Gordon@rockets.utoledo.edu (D.M.G.); Stephen.Hong@rockets.utoledo.edu (S.H.H.); 2 Department of Pharmacology and Nutritional Sciences, University of Kentucky College of Medicine, 760 Press Avenue, Healthy Kentucky Research Building, Lexington, KY 40508, USA; Zachary.Kipp@uky.edu

**Keywords:** autofluorescence, heme oxygenase, HO-1, biliverdin reductase, BVRA, albumin, bilirubin, PPAR, nuclear receptor, mutagenesis analysis

## Abstract

Recent work has shown that bilirubin has a hormonal function by binding to the peroxisome proliferator-activated receptor-α (PPARα), a nuclear receptor that drives the transcription of genes to control adiposity. Our previous in silico work predicted three potential amino acids that bilirubin may interact with by hydrogen bonding in the PPARα ligand-binding domain (LBD), which could be responsible for the ligand-induced function. To further reveal the amino acids that bilirubin interacts with in the PPARα LBD, we harnessed bilirubin’s known fluorescent properties when bound to proteins such as albumin. Our work here revealed that bilirubin interacts with threonine 283 (T283) and alanine 333 (A333) for ligand binding. Mutational analysis of T283 and A333 showed significantly reduced bilirubin binding, reductions of 11.4% and 17.0%, respectively. Fenofibrate competitive binding studies for the PPARα LBD showed that bilirubin and fenofibrate possibly interact with different amino acid residues. Furthermore, bilirubin showed no interaction with PPARγ. This is the first study to reveal the amino acids responsible for bilirubin binding in the ligand-binding pocket of PPARα. Our work offers new insight into the mechanistic actions of a well-known molecule, bilirubin, and new fronts into its mechanisms.

## 1. Introduction

The peroxisome proliferator-activated receptors (PPARs) are a class of nuclear receptors that have become major targets for addressing various metabolic disorders, including lipid dystrophies and diabetes [1]. PPARs are nutrient receptors that have various selective and promiscuous ligands, including fatty acids and eicosanoids. There are three major isoforms of PPARs: PPARα, PPARβ/δ, and PPARγ. PPARα and PPARβ/δ induce genes to regulate lipid uptake and metabolism [1,2,3]. PPARγ, which is additionally stimulated by thiazolidinediones (TZDs; rosiglitazone, pioglitazone) [4], induces transcription of genes involved in fat storage and lipogenesis [1,2,5]. These nuclear receptors play key roles in regulating cellular metabolism, which results in phenotypic changes such as improved glucose storage and reduced serum lipids. Therefore, understanding the ligand-binding capability of these nuclear receptors is important in appreciating the pharmacokinetics of newly developed compounds that target obesity and diabetes. In our previous work, we found structural similarities between bilirubin, the red blood cell metabolite, and known ligands of PPARα [3]. Furthermore, clinical phenotypes associated with mildly elevated bilirubin levels are associated with reduced adiposity, suggesting an interaction between PPARα and bilirubin. We have previously addressed this binding interaction by quantifying the binding modalities between bilirubin and PPARα [6], which was achieved via fluorescent-based assays similar to the well-known bilirubin albumin autofluorescence.

The autofluorescence phenomenon is only demonstrated when bilirubin was bound to protein (albumin), and either alone did not display these properties [7,8,9,10]. When bilirubin binds albumin and is excited by light, it undergoes a conformational change (Z → E configuration). This configuration, now photobilirubin/lumirubin, allows for greater mobility of the pyrrole rings and subsequent emission behavior [11,12,13]. Utilizing this property of bilirubin to fluoresce when bound to proteins, aside from albumin, is a new concept to explore binding interactions. In this study, we explore the binding capability of bilirubin to the PPARα ligand-binding domain (LBD) using bilirubin’s autofluorescent properties. Various studies have explored the binding capabilities of known ligands to PPARs using fluorescence or radioactive means of measurement [14,15,16]. Other studies have shown that bilirubin induces fluorescent properties by interacting with the fluorescent protein UnaG protein from eel muscle [17,18]. Our study here focused on the intrinsic properties of bilirubin to autofluoresce when excited by light and bound to protein, specifically PPARα and mutations in amino acids of the LBD that may be responsible for bilirubin binding. Ultimately, investigation of this interaction will highlight the role of bilirubin as a metabolic hormone to activate nuclear receptors, resulting in phenotypic improvements in metabolism (discussed further in our extensive review [19]).

We found that bilirubin bound to albumin autofluoresces in a dose–response relationship and show bilirubin bound to PPARα autofluoresces in a similar manner. We present bilirubin’s binding capacity with specific amino acids in the PPARα LBD and show that this can be quantitated based on its autofluorescence.

## 2. Materials and Methods

### 2.1. Reagents

Phosphate-buffered saline (PBS) (Amresco, Solon, OH, USA), bovine serum albumin (BSA) (Fisher Scientific, Hampton, NH, USA), unconjugated bilirubin (Frontiers Scientific, Logan, UT, USA), and dimethyl sulfoxide (DMSO) (MP Biomedicals, Solon, OH, USA).

### 2.2. Cell Culture

Human Embryonic Kidney 293 (HEK293) cells were routinely cultured and maintained in Dulbecco’s Modified Eagle Medium (DMEM) containing 10% FBS with 1% penicillin-streptomycin.

### 2.3. Full-Length Histidine-Tagged PPAR Construction

Full-length histidine-tagged mouse PPARα and PPARγ sequences were amplified with designated primer for restriction enzymes as listed in the table below using the KOD Hot Start DNA polymerase kit (Millipore Sigma, Burlington, MA, USA). A 1% agarose gel verified the presence of a single band of the PPAR at ~1.5 kb, which was purified using the Qiagen Gel extraction (Qiagen Biotechnology, Hilden, Germany). Primer sequences for cloning PPARα were forward-containing BamHI restriction sites (5′-CGGGATCCGATGGTGGACACAGAGAGCC-3′) and reverse-containing XbaI restriction sites (5′-GCTCTAGACTCTTCATCCCCAAGCGTAG-3′). Primer sequences for cloning PPARγ were forward-containing KpnI restriction sites (5′-GGGGTACCTTATGGGTGAAACTCTGGGAGAT-3′) and reverse-containing XbaI restriction sites (5′-GCTCTAGAAGAAGGAACACGTTGTCAGC-3′). The PCR products and the pcDNA6/His A vector (Invitrogen, Carlsbad, CA) were digested with the appropriate restriction enzymes described in the table below for each respective isoform overnight at 37 °C. A 1% agarose gel verified the presence of a single band at approximately 5.2 kb for the pcDNA6HisA vector, which was purified using the Qiagen Gel extraction kit. The PPARα or PPARγ PCR products were purified using the Qiagen PCR purification kit (Qiagen Biotechnology, Hilden, Germany). Ligation of the pcDNA6/HisA vector and the PPARα or PPARγ PCR products was achieved using a Rapid Ligation kit (Thermofisher Scientific, Waltham, MA, USA) for 1 h at room temperature. The ligation product was transformed into DH5α cells (Invitrogen, Carlsbad, CA, USA). Colonies were grown on 0.1% ampicillin fortified agarose plates overnight in a 37 °C non-CO_2_ incubator. Individual colonies were selected and grown in LB broth with 0.1% ampicillin. The plasmid from selected colonies was isolated and purified using the Qiagen Mini-Prep kit (Qiagen Biotechnology, Hilden, Germany). Sequencing of the plasmids was performed using primers T7 forward (5′-TAATACGACTCACTATAGGG-3′) and BGH Reverse (5′-TAGAAGGCACAGTCGAGG-3′). Successfully cloned colonies were grown in 500 mL of LB broth plus 0.1% ampicillin, and plasmids were extracted and purified using the Denville Spinsmart Maxi kit (Denville, Swedesboro, NJ, USA).

### 2.4. PPARα Ligand-Binding Domain Mutagenesis

To determine the specificity of bilirubin-binding capacity with PPARα in the ligand-binding domain, mutant proteins were created to target the sites previously shown to possibly hydrogen bond with PPARα for optimal binding [3]. The mutant PPARα A333G, M330G, and T283G were generated using QuikChange site-directed mutagenesis kit with the PPARα-pcDNA6/HisA plasmid according to the manufacturer’s protocol (Stratagene, La Jolla, CA, USA). Primers for the mutant binding site were created using the QuickChange Primer Design program available on the Agilent website. The mutations were achieved using the QuikChange Lightning Site-Directed Mutagenesis Kit (Agilent, Santa Clara, CA, USA). Plasmids were transfected into XL 10-Gold Ultra Competent E. Coli cells and grown on a 1% ampicillin agar plate. Colonies were grown on 0.1% ampicillin fortified agarose plates overnight in a 37 °C non-CO2 incubator. Individual colonies were selected and grown in L.B. broth with 0.1% ampicillin. The plasmid from selected colonies was isolated and purified using the Qiagen Mini-Prep kit. Confirmation of successful mutation was achieved via sequencing with Eurofins Genomics.

### 2.5. Purification of PPAR Proteins

Transient transfections were performed using GeneFect (Alkali Scientific Inc., Pompano Beach, FL, USA) in the HEK293 cells for 48 h. Cells were harvested via centrifugation and resuspended in 200 μM HEPES. The His-Tagged proteins were extracted using the HisLink™ Protein Purification Resin (Promega, Madison, WI, USA). Proteins were subsequently dialyzed overnight to remove excess elution compounds. The proteins were quantified using the BCA Protein Assay Kit (Thermofisher Scientific, Waltham, MA, USA). To confirm that the correct protein was purified, protein extracts were resolved by SDS polyacrylamide gel electrophoresis and electrophoretically transferred to Immobilon-FL membranes. Membranes were blocked at room temperature for 1 h in Odyssey Blocking buffer (LI-COR Biosciences, Lincoln, NE, USA). Subsequently, the membrane was incubated overnight at 4 °C with PPARα (sc-1982), PPARγ (sc-7273), or His-Probe Antibody (sc-8036). After three washes in TBST (TBS plus 0.1% Tween 20), the membrane was incubated with an infrared anti-goat (IRDye 800, green) or anti-mouse (IRDye 680, red) secondary antibody labeled with IRDye infrared dye (LI-COR Biosciences) (1:15,000 dilution in TBS) for 2 h at 4 °C. Immunoreactivity was visualized and quantified by infrared scanning in the Odyssey system (LI-COR Biosciences, Lincoln, NE, USA).

### 2.6. General Autofluoresce Assay Setup

In a black flat-bottom 96-well plate, stock BSA (final concentration 50 μM), stock PPARα (final concentration 3–7.8 μM), or PBS was added to the wells. Next, the test compound bilirubin was mixed into a well, repeated in triplicate, with the PBS, BSA, or PPARα. Once all compounds were added to the plate, the plate was protected with foil to avoid light degradation. Excitation and emission spectra of the samples were recorded using the top-read SpectraMax Plate Reader (Molecular Devices, San Jose CA, USA). The samples were read in 5 nm steps with both excitation and emission filters in use. The excitation spectrum was recorded from 300 to 495 nm. Once the maximal emission for bilirubin bound to the albumin or PPARα was recorded, then the wavelength of the peak value was set as the maximal excitation value and used for the excitation wavelength for the subsequent emission spectrum from 495 to 700 nm.

### 2.7. Statistics

The data were analyzed via Prizm 8 GraphPad Prism version 8.00 for Mac (GraphPad Software, La Jolla, CA, USA) using analysis of variance combined with Tukey’s post-test to compare pairs of group means or unpaired *t-*tests. Additionally, one-way ANOVA with the least significant difference post hoc test was used to compare mean values between multiple groups. Results are shown as the mean ± S.E.M. *p* values of 0.05 or smaller were considered statistically significant.

## 3. Results

### 3.1. Autofluorescent Properties of Bilirubin and Biliverdin When Bound to Albumin

**Figure 1:**
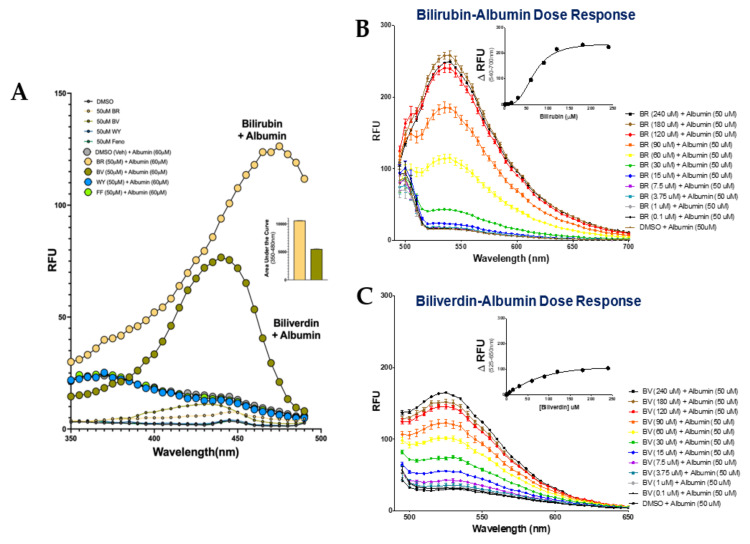
Bilirubin and biliverdin autofluoresce when bound to albumin. (**A**) Bilirubin and biliverdin binding to albumin emission at 520 nm (*n* = 3). (**B**) Bilirubin-bound albumin dose–response curves excited at 465 nm (*n* = 3); ΔRFU was calculated at 540–700 nm. (**C**) Biliverdin-bound albumin dose–response curves excited at 440 nm (*n* = 3; ΔRFU was calculated at 525–650nm. BR, bilirubin; BV. biliverdin; WY, WY 14,643; FF, fenofibrate; DMSO, vehicle.

Studies have shown that bilirubin autofluoresces when bound to albumin [9,10,11]. This relationship has been studied for decades due to albumin’s regulation of free small molecule and protein concentration in the plasma. Work on the bilirubin–albumin interaction has shown that bilirubin emits its maximum fluorescence at 520 nm [9]; therefore, we set our excitation spectrum to record fluorescence at 520 nm. As previously shown, our results revealed minimal fluorescence of bilirubin alone in the absence of albumin (Figure 1A). In the presence of albumin, there is a significant shift in relative fluorescence units (RFU), with the maximum emission reported at an excitation wavelength of 465 nm, as reported by us and others [9,20]. Biliverdin, in the presence of albumin, also has a shift in RFUs compared to no albumin, indicating that it is also binding to albumin but with a peak at 440 nm. The bilirubin-bound albumin had a 2-fold area under the curve (AUC) greater autofluoresce when compared to biliverdin-bound albumin. The excitation wavelength for bilirubin-bound albumin was set at 465 nm for subsequent emission spectra. The emission spectra in the 350–480 nm range for bilirubin with and without albumin recapitulates the previously published data that albumin is necessary for bilirubin to autofluoresce [9,20]. We also found that PPARα ligands WY 14,643 and fenofibrate had no fluorescent activity with or without albumin.

We next determined the binding of bilirubin to albumin via fluorescence using the 465 nm excitation measuring in the 540–700 nm as previously shown [9]. An increasing dose of bilirubin was used in the presence of albumin to determine the maximal shift in autofluorescence. A Gaussian curve was fit to each spectrum, revealing a dose-dependent increase in fluorescence (Figure 1B). As in previous studies, the maximum fluorescence (F_max_) was recorded and defined as the peak fluorescence intensity within the emission spectra for each condition [9]. To create a dose–response curve, ΔRFU values were calculated by subtracting the F_max_ recorded with bilirubin alone at a given concentration from the F_max_ at the same concentration but in the presence of albumin. These values were used as the ΔRFU for subsequent analysis to determine the specific binding. For affinity analysis, the specific binding F_max_ per condition was plotted against the concentration of bilirubin, and a non-linear line was fit to the graph. The disassociation constant (K_d_) was calculated using the fitted line and was determined to be the concentration in which half of the maximum fluorescence was achieved. A line fit to the F_max_ at each concentration revealed a K_d_ value of 11.10 μM for bilirubin-bound albumin. To further analyze the biliverdin-bound albumin autofluorescence, we measured fluorescence using the 440 nm excitation over the 525–650 nm spectra as these were the peak values for biliverdin in Figure 1A. The results in Figure 1C show a dose-dependent increase in fluorescence for biliverdin bound to albumin.

### 3.2. Bilirubin Fluoresces when Bound to PPARα

**Figure 2:**
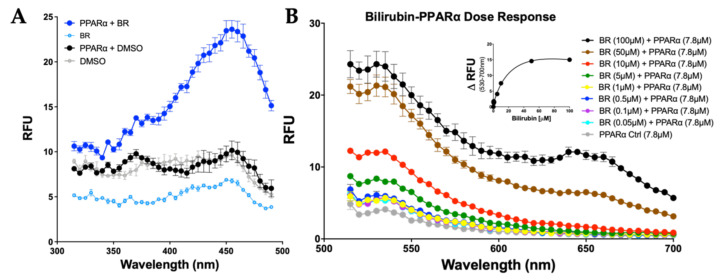
Bilirubin bound to PPARα autofluoresces. (**A**) Bilirubin binding to PPARα was measured at emission at 540 nm (*n* = 3). (**B**) Bilirubin-bound PPARα dose–response curves excited at 455 nm (*n* = 3); ΔRFU was calculated at 530–700 nm. BR, bilirubin; DMSO, vehicle.

Many studies have exploited the property of bilirubin to autofluoresce when bound to albumin, and recently we showed that this fluorescent capability could be extended to study bilirubin’s interactions with other proteins [20]. Given that bilirubin was shown to bind directly to PPARα, we wanted to determine at what level autofluoresce excitation occurs for bilirubin-bound PPARα. We used purified histidine-tagged PPARα (His-PPARα), bilirubin and vehicle (DMSO) alone, and a combination of PPARα and bilirubin and measured emission set at 520 nm. The maximal excitation fluorescence of bilirubin bound to PPARα was found to be at 450 nm (Figure 2A). Comparing the PPARα-bound bilirubin to albumin-bound in Figure 1A, there was a shift in PPARα-bilirubin from maximal fluorescence of 465 to 455 nm for PPARα. We incubated PPARα with increasing amounts of bilirubin and recorded the emission at its max peak. Therefore, we set up the excitation of the bilirubin-PPARα complex at 455 nm and measured the 530–700 nm wavelength. Our results showed a significant increase in bilirubin-induced fluorescent activity with an increasing bilirubin concentration (Figure 2B). The ΔRFU for each concentration was plotted against the concentration of bilirubin, with a line of best fit, revealing a K_d_ value of 5.13 μM. Of importance, the level of RFU autofluorescence observed in albumin-bound bilirubin compared to PPARα-bound bilirubin, the protein level was significantly higher at 50 μM albumin compared to 7.8 μM PPARα. Overall, these data show that bilirubin directly binds to PPARα. However, whether bilirubin binds directly to the LBD cannot be determined from these data. Therefore, we performed site-specific mutagenesis to determine binding areas.

### 3.3. Bilirubin Requires Distinct Amino Acids to Maximize Binding to PPARα

**Figure 3:**
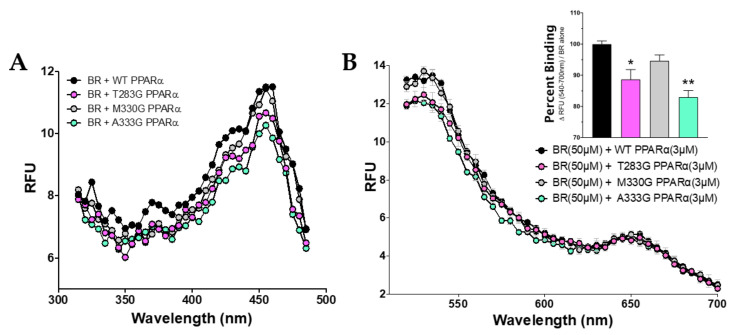
Amino acids in the PPARα ligand-binding domain that bilirubin binds to induce autofluoresce. (**A**) Bilirubin binding to WT PPARα and ligand-binding mutants T283G PPARα, M330G PPARα, and A333G PPARα emission at 540 nm (*n* = 3). (**B**) Bilirubin-bound PPARα WT and mutants excited at 455 nm (*n* = 3); percent binding = [(ΔRFU was calculated at 540–700 nm)/(BR alone)*100]. BR, bilirubin.

PPARα has a single low-fidelity ligand-binding pocket. Our previously published in silico docking analysis revealed several predicted residues that bilirubin might interact in the PPARα LBD by hydrogen bonding to stabilize binding [3]. Therefore, we explored how mutating the predicted hydrogen bonding sites of interaction might affect bilirubin’s binding capacity. Mutations were made in our histidine-tagged WT PPARα (His-PPARα) vector via the substitution of glycine at the following sites: Threonine 283 (T283G), methionine 330 (M330G), and alanine 333 (A333G). We measured the emission set at 540 nm for WT PPARα and mutants. The results show a reduction in the maximal fluorescence between the mutants and WT (Figure 3A). The maximal excitation fluorescence of bilirubin bound to PPARα was found to be at 455 nm, which was used in the next analysis to determine which amino acids are bound by bilirubin. Using the WT PPARα and mutants at an excitation of 455 nm, the wavelengths were measured in 530–700 nm spectra, and the ΔRFU was calculated for subsequent analysis to determine the specific binding (Figure 3B). There was a significant decrease in bilirubin-induced fluorescence for PPARα mutants T283G (vs. WT, *p* < 0.05) and A333G (vs. WT, *p* < 0.01). There was no significant decrease in fluorescence between the WT and PPARα mutant M330G (*p* = 0.3673).

### 3.4. Specific Binding of Bilirubin to PPARs

**Figure 4:**
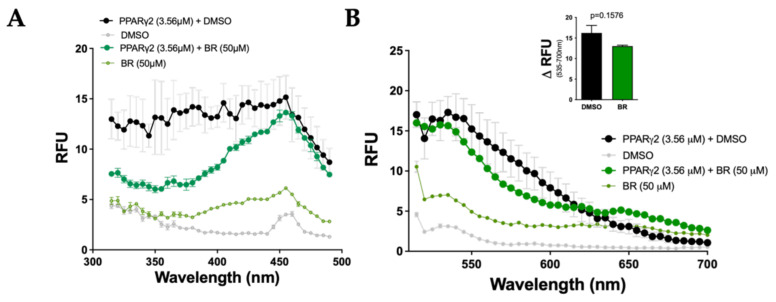
Bilirubin does not autofluoresce with PPARγ2. (**A**) Bilirubin binding to PPARγ2 was measured at emission at 540 nm (*n* = 3). (**B**) Bilirubin and PPARγ2 were excited at 455 nm and spectra were measured at 510 to 700 nm (*n* = 3); ΔRFU was calculated at 535–700 nm. BR, bilirubin; DMSO, vehicle.

**Figure 5:**
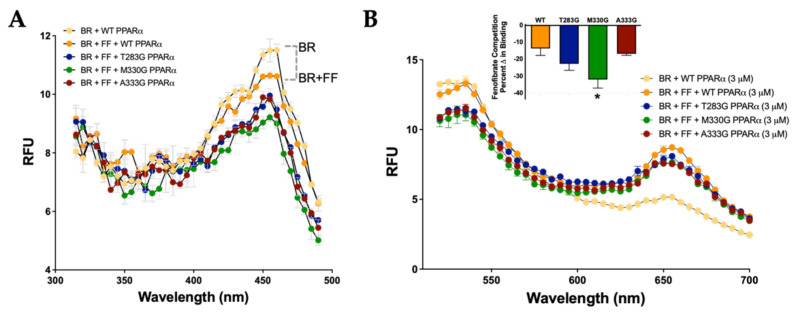
Bilirubin competes with fenofibrate for binding the PPARα ligand-binding domain. (**A**) Bilirubin binding to WT PPARα and ligand-binding mutants T283G PPARα, M330G PPARα, and A333G PPARα emission at 540 nm (*n* = 3). (**B**) Bilirubin-bound PPARα WT and mutants excited at 455 nm (*n* = 3); percent binding = [(ΔRFU was calculated at 540–700 nm)/(BR alone)*100] and then BR-BR + FF for each mutant. BR, bilirubin; FF, fenofibrate.

Previously, we have shown that bilirubin did not bind PPARγ [20], which here we wanted to determine whether there might be an interaction that induces autofluoresce. Measurement of the emission at 540 nm or excitation at 455 nm and analysis of the 510 to 700 nm spectra for bilirubin and PPARγ showed no autofluoresce as was observed with PPARα (Figure 4A,B). We have previously shown that PPARα-bound bilirubin competed for the LBD with fenofibrate [20]. To determine how bilirubin and fenofibrate were affected by the site-specific mutants during the competition binding, we performed the same assay described in Figure 3. The results show that bilirubin and fenofibrate compete for the PPARα LBD and that the Met330 site is more relevant for fenofibrate binding (Figure 5A,B).

## 4. Discussion

The concept that bilirubin may function as a hormone by directly binding to the PPARα nuclear receptor is a new concept, shifting the thinking about this ‘old molecule’ [19,21,22,23]. Recent work using bilirubin nanoparticles has shown that this is a fat-busting hormone that reduces body weight and adipocyte size in obese mice [20] and fatty liver disease while not causing liver dysfunction [6]. Studies have revealed an association with lower incidences of cardiovascular events and metabolic syndrome in patients with mildly elevated bilirubin levels [24,25]. These posit that there is more to be known about this old molecule.

In silico docking analysis from our previous work revealed potential sites that bilirubin may interact in the PPARα ligand-binding pocket [3]. Here, we wanted to determine whether these sites were essential by mutating the amino acid sites (T283G, M330G, and A333G) to determine they are necessary for the interaction of bilirubin with the PPARα LBD. Our results reveal compelling data that detail interactions between PPARα and bilirubin via their autofluorescent emission upon excitation in the presence of PPARα versus with either alone. Our previous work showed that bilirubin induced transcriptional activity of the GAL4-PPARα LBD construct with an EC_50_ of 9.0 μM [6], which in this study using a different technique found that the K_d_ value of bilirubin-bound PPARα was 5.13 μM. We also found that bilirubin did not drive the transcriptional activity of the other PPAR isoforms, PPARγ or PPARβ/δ [6]. Here, we found that autofluoresce was not observed between PPARγ and bilirubin, indicating no binding, which supports our previous finding that bilirubin did not drive the transcriptional activity of this isoform. The results from this study reveal the importance of the amino acid residues for the predicted binding sites. We found that mutational analysis of T283 and A333 of the PPARα LBD showed significantly reduced bilirubin binding, reductions of 11.4% and 17.0%, respectively. We also found that fenofibrate and bilirubin compete for the PPARα binding site and that the Met330 site might be essential for fenofibrate binding. A study by Yamamoto et al. confirmed that Met330 is a critical site for fenofibrate binding in the PPARα LBD to induce transcriptional activity [26]. Their investigation also showed that another PPARα ligand, pemafibrate, was not affected by mutational analysis of the Met330 site. These suggest that the differential control of specific gene pathways might depend on ligand binding with particular amino acids that cause a conformation change in the PPARα structure that induces pathway-specific patterns. Furthermore, bilirubin and its light-reactive form, lumirubin, have recently been shown by others to activate PPARα transcriptional activity in HepG2 human hepatocytes [27]. Their findings showed that bilirubin and lumirubin had diverse gene regulatory patterns, suggesting that different amino acids might regulate the binding of these molecules in the PPARα LBD.

The link between bilirubin and cardiometabolic disease has been suggested to exist via the interaction of bilirubin and PPARα [21]. While this study confirms a direct interaction between bilirubin and amino acids in the LBD, previous studies have shown bilirubin to positively influence metabolic status via PPARα. Bilirubin’s role in metabolism is poorly understood. Our previous studies showed a loss in bilirubin mediated weight loss in the absence of PPARα [3]. There may be interactions of bilirubin with other proteins outside of PPARα. However, our recent study showed that bilirubin-induced transcriptome responses were 95% PPARα dependent [28], indicating that this nuclear receptor might be primarily responsible for its induction of gene transcripts. However, more work is needed to build upon this conclusion.

PPARα has been shown to alter gene expression for various cellular functions but is widely studied for its impact in increasing lipid metabolism. PPARα hepatocyte-specific knockout mice have a characteristically increased hepatic lipid storage [29], and ob/ob leptin-deficient obese mice have reduced plasma bilirubin and hepatic PPARα [30]. Adipocytes and hepatocytes treated with bilirubin had a significantly lower level of lipid accumulation compared to the control-treated cells [3,31]. The bilirubin-induced regulation of PPARα may help to understand clinical phenotypes in patients with lower bilirubin levels [19]. Mutations of the PPARα LBD reinforce our previous in silico-predicted amino acid residues that might stabilize binding with bilirubin [3]. The loss of this stable interaction confirms bilirubin’s conformational stringency in binding to PPARα. This could explain why biliverdin, the “prodrug” of bilirubin, did not show strong binding to PPARα [3]. Hence, a hepatocyte-specific knockout of biliverdin reductase A (BVRA), the enzyme responsible for producing bilirubin [32], causes severe hepatic steatosis and glucose intolerance [33]. Mice with an adipose-specific BVRA KO showed increased adipocyte size and reduced mitochondria [34]. On the other hand, bilirubin nanoparticle treatment in obese mice decreased white adipose tissue (WAT) size and increased mitochondria number [20]. These imply that possibly inducing heme oxygenase or BVRA might improve adiposity by increasing plasma bilirubin [35]. This concept is supported in a study showing that high-aerobic-capacity running rats had significantly higher plasma bilirubin and increased hepatic BVRA and PPARα than the low-running-capacity obese animals [36].

## 5. Conclusions

The data presented here clearly reveal the interactions of bilirubin with the PPARα LBD. These interactions reveal the potential of bilirubin in modulating cellular processes and ultimately enhancing metabolic potential. Studies have indicated lesser incidences in cardiovascular events and metabolic syndrome in patients with mildly elevated bilirubin levels [24,25], and mice with hyperbilirubinemia are protected from adiposity [6,37]. Further studies are needed to highlight the effect of bilirubin in mediating metabolic potential compared to other known ligands of PPARα such as the fibrates. Unveiling the role of bilirubin as a metabolic hormone posits that the old molecule is a potential new agent for addressing the metabolic syndrome.

## Data Availability

The data presented in this study are available on request from the corresponding author.
